# Septic Arthritis in Rheumatology: Review of Five Cases

**DOI:** 10.4021/jocmr2009.08.1254

**Published:** 2009-08-04

**Authors:** Azura Dina Muhayidina, Mohd Shahrir Mohamed Said

**Affiliations:** a Medical Department UKM Medical Center, Malaysia; b Rheumatology Unit, Medical Department UKM Medical center, Malaysia

## Abstract

This report illustrates five cases of patients admitted to medical ward in HUKM, diagnosed and treated as septic arthritis over the course of two months. Their age ranged from 32 to 67 years old with one patient had history of monoarticular pain and the other four had polyarticular pain. Two of these patients had pre-existing joint disease, namely gouty arthritis and rheumatoid arthritis, and another patient with background history of mixed connective tissue disease on long term steroid therapy. The diagnosis of septic arthritis was made mainly from clinical assessment, supported by synovial fluid assessment and blood investigations. All patients received minimum of two weeks intravenous antibiotic followed by one month course of antibiotic. All of them had arthrocentesis for diagnostic and therapeutic purposes and two had laparoscopic arthroscopy with wash out done.

**Keywords:**

Septic arthritis; Arthrocentesis; Arthroscopy

## Introduction

Bacterial septic arthritis is one of the rheumatological emergencies that require prompt diagnosis and treatment due to its rapid destructive nature leading to high morbidity and mortality rate. The annual incident is approximately 2-5/100000 in general population and increases to 28-38/100000 in those with pre-existing inflammatory arthritis [1]. Polyarticular involvement occurs in 15 to 20% of all septic arthritis cases and carries 30% mortality rate as compare to 4% mortality rate in monoarticular involvement [2].

The diagnosis can be difficult, especially in patients with underlying inflammatory illness, as it can be mistaken as flare of the underlying illness. Diagnosis is usually based on clinical assessment of an experienced physician, supported by synovial fluid assessment for culture, gram stain and white cell count. Leucocytosis and raised inflammatory markers are not diagnostic but support diagnosis of septic arthritis.

The commonest cause of septic arthritis in sexually active young adults is N. Gonorrhoeae (i.e 75% of cases) [3, 4]. In older adults, Staphylococcus aureus account for almost 80% of patients with polyarticular septic arthritis and 62% with monoarticular septic arthritis [1, 2, 5-7]. Streptococcus species is a causative organism for about 4% of polyarticular septic arthritis cases and up to 19% of monoarticular septic arthritis cases [1, 2, 5-7]. Gram negative organisms are usually seen in immunocompromised patients such as in the elderly, those with systemic lupus erythromatosus and malignancy [2, 6, 7].

Treatments include appropriate antibiotic therapy and drainage of the affected joint(s). Optimization of treatment for other co-morbidities such as diabetes mellitus, removal of joint prostheses, discontinuation of immunosuppressant, and mobilization therapy are all equally important.

## Cases characteristics

This paper reports five patients with various medical background presented with either monoarticular or polyarticular joint pain and swelling. Only four of them presented with high grade fever of varying duration. Diagnoses of septic arthritis was made mainly on clinical ground for all cases, supported by synovial fluid examination and other supportive features from blood investigations such as leucocytosis, raised ESR and C-reactive protein. The first two cases were diagnosed to have reactive arthritis due to Chlamydia pneumoniae infection and possible infective endocarditis respectively but later revised to septic arthritis. All patients received intravenous antibiotics; choice differs based on individuals background and suspected organism. Decisions to proceed with arthrocentesis or arthroscopy were based on site of joint involvement and patients response to medical therapy. The details on each case are as described.

### Case 1

A 32 year old man presented with five days history of high grade fever associated with severe non flitting polyarthritis involving right shoulder, elbow, hip and ankle, bilateral knees and bilateral first metarcapophalangel joints. The right knee and ankle were markedly swollen and red. He denied any other symptom to suggest respiratory, gastrointestinal or connective tissue disease. However, he gave history of recent unprotected sexual intercourse with multiple partners during his business trips. He had no history of sore throat, genital ulcer, dysuria, abnormal urethral discharge or rash.

Upon presentation, he was ill looking but hemodynamically stable and was afebrile. There was no sign of rheumatic fever or sexually transmitted disease and other systems examination was unremarkable. The right knee effusion was tender, warm and erythematous with marked reduction in range of movement. The other affected joints were not swollen, but tender and warm.

His blood investigations revealed leucocytosis with raised inflammatory markers. (WBC 21.1 x 10^9^/L, HB 15.8 g/dL, PLT 332 x 10^9^/L, ANC 17.5 x 10^9^/L, ESR 76 mm/hr, CRP 11 mg/dl). His chest radiograph showed right mid and lower zone air bronchogram and he was started on intravenous augmentin and azithromycin. Differential diagnosis of acute rheumatic fever was excluded as there was no further evident to support this diagnosis and sexually transmitted disease screening was negative. Blood culture was negative and sputum culture was not available. Chlamydia pneumoniae IgM serology was positive suggestive of respiratory Chlamydia infection.

After a few days, he developed worsening right knee effusion, high grade temperature (38 ^o^C) and elevated C-reactive protein level (23 mg/dl). Intravenous Augmentin was changed to Ceftriaxone in view of possible gonorrheal septic arthritis. Diagnostic right knee aspiration revealed 30 ml of turbid synovial fluid. Synovial fluid examination showed packed full white blood cells (> 10 polymorphs/HPF), but no organism or acid fast bacilli seen and the culture was negative. Right arthroscopy with wash out was done. Subsequently, he recovered well and completed two weeks of IV Rocephine 1g OD. He was discharged home with one month oral Cefuroxime 250mg BD.

### Case 2

A 49 years old man with St Judes mechanical aortic valve and hyperlipidemia, presented with painful right second metartasophalangeal, left knee, left ankle and right hip pain for a week. The second left metartasophalangeal and left knee were swollen and it was associated with persistent high grade fever. There was no history of recent dental surgery, fall or trauma.

On examination, there was no sign of infective endocarditis. The second left metartasophalangeal and left knee joints were warm, erythematous, swollen and tender, associated with reduced range of movement. There was also evident of superficial abrasion and skin break overlying the second toe. The other affected joints were not swollen or erythematous, but tender and warm. The rest of the examination was unremarkable.

His initial blood investigations showed WBC count of 9.4 x 10^9^/L and C-reactive protein was elevated to 9.8 mg/dL. He was admitted for further workout to exclude infective endocarditis and was started on oral cloxacillin in view of skin infection.

However, after a week, his joints became more tender, erythematous and swollen. His blood cultures were negative and echocardiogram showed no vegetation. Radiographs of the affected showed soft tissue swelling, but otherwise normal. The diagnosis was revised to polyarticular septic arthritis and left knee joint aspiration revealed turbid colored fluid. There were numerous white blood cells and red cells (polymorphs 232/mm^3^, lymphocytes 136/mm^3^ and red blood cell 600/mm^3^), but the Ziehl Nielsen stain and culture was negative. He recovered well and discharged after two weeks with a month supply of oral cefuroxime 250 mg BD.

### Case 3

A 60 years old woman was first seen at our clinic with two years history suggestive of rheumatoid arthritis involving proximal interphalangeals, metarcarpophalangeals, wrists, elbows, knees and ankles joint.

Two weeks prior to presentation, her wrists, knees, ankles and shoulders were swollen and she was having high grade fever with chills and rigors.

Musculoskeletal examination revealed symmetrical polyarthritis with tenosynovitis of the wrists, shoulders, knees and ankles. The right knee was significantly more swollen and tender.

Her blood work up showed the expected leucocytosis and raised inflammatory markers. (WBC 11.2 x 10^9^/L, HB 10.6 g/dL, PLT 358 x 10^9^/L, ANC 7.3 x 10^9^/L, ESR 116 mm/hr, CRP 7.62 mg/dl). X-rays of the hands and feet joints generally showed evident of juxtaarticular osteopenia, but no narrowing of joint spaces or marginal erosions. However, there were reduced joint spaces and subchondral sclerosis seen at bilateral knee joints.

Purulent synovial fluid was aspirated from the right knee and normal saline wash out was done. The synovial fluid culture was negative and there was no organism seen on gram stain examination. She was started on IV Unasyn 750mg TDS and her fever and pain gradually improved. After a week, intravenous hydrocortisone 50 mg TDS and Oral sulphasalazine 500mg OD were started to reduce inflammatory process of her other joints.

She had swift recovery and discharged home after two weeks with oral Unasyn 375 mg BD for a month, oral prednisolone 15 mg OD and Oral sulphasalazine 500 mg BD.

### Case 4

A sixty years old man presented with multiple joints pain, involving proximal interphalangeal joints, metarcapophalangeal joints, wrists, shoulders, neck, ankles and knees for four months associated with progressive restriction of movements. This was also associated with early morning stiffness, malaise, lethargy, loss of weight and loss of appetite.

On admission, his complaint of a month history of unresolved fever associated with severe pain and swelling of both shoulders and knees, resulted in total dependence on all activities of daily living. Two weeks prior to that, he visited a local healer whom performed a traditional method to relieve pain by compressing his toes with a piece of wood. Following this, he developed left first and second nail bed abscess resulted in a visit to another hospital for removal of the nails.

On examination, he was very ill looking and febrile. Both shoulders and knee joints were swollen, tender, warm and erythematous. There was no evidence of chronic rheumatoid arthritis joint changes or extraarticular involvement. However, the left first and second toe was gangrenous. The differential diagnoses at this juncture include flare of rheumatoid arthritis, polyarticular septic arthritis and polyarticular gouty arthritis.

Blood investigations showed raised white cells count, ESR and C-reactive protein. (WBC 27.5 x 10^9^/L, HB 9.0 g/dL, PLT 484 x 10^9^/L, ANC 26.1 x 10^9^/L, ESR 76 mm/hr, CRP 17.27 mg/dl). His rheumatoid factor was negative and serum uric acid was 196 umol/L. Radiograph of the right shoulder showed reduced joint space with evident of soft tissue swelling and air in the joint space, suggestive of an abscess. The feet, hands and left shoulder x-ray were not remarkable, and knee x-rays showed minimal osteophytes in keeping with degenerative changes.

Joint aspirate from shoulders and knees showed turbid purulent fluid from all affected joints with packed filled full of polymorphs seen under the microscope. Gram stain and Ziehl Nielsen stain was negative. Cultures from the synovial fluid and blood were negative as well.

He required multiple aspiration of the pus and due to unresolved fever, IV Augmentin and IV Ciprofloxacin were escalated to IV Meropenam 1g TDS. After almost a month, his bilateral knee swelling and pain resolved. However, his shoulders swelling persisted and ultrasound of the joint showed evident of multiple tendons tear. A repeat right shoulder synovial fluid aspirate showed straw colored fluid with no polymorphs, but negatively befringents crystal was seen, suggestive of gouty arthritis.

He was immediately started on oral colchicine and prednisolone in addition to the antibiotic. He was also referred to orthopaedic team for tendon repair and currently undergoing physiotherapy.

### Case 5

A 67 years old woman, premorbidly bed ridden due to stroke was brought in from nursing home after complaining of left knee pain and swelling for a week. She denied any history of fever, fall or trauma. Her other medical illnesses include mixed connective tissue disease, diabetes mellitus, hypertension and chronic atrial fibrillation. She was also on long term oral prednisolone 15 mg OD for autoimmune hemolytic anemia and vasculitis.

On admission, she was septic looking and in severe pain. The left knee was swollen, tender, warm and erythematous, extending proximally to mid thigh. There were multiple inguinal lymph nodes palpable. Other systems examinations were unremarkable.

Her total WBC was 19.3 x 10^9^/L with neutrophilia of 18.2 x 10^9^/L. Her ESR was 86 mm/hour and C-reactive protein was 16.8 mg/dL. Ultrasound of the left knee showed hypoechoic collection at the suprapatella region with some internal echos and septation within the lesion. The collection was seen within the muscular striation and appears to be continous with the subpatellar knee joint region. Left knee x-ray showed soft tissue swelling at suprapatellar region with reduced joint space and osteophytes. Aspiration from the joint revealed thick, turbid fluid. There were more than 500 white blood cells/mm^3^ and more than 500 red blood cells/mm^3^ in the synovial fluid and culture grew Pseudomonas Species. Blood culture grew Bacillus Species and urine culture grew Klebsiella ESBL. She responded well with intravenous Meropenam and oral steroid was reintroduced slowly.

She underwent arthroscopic knee wash out at day 14 of admission and repeat synovial fluid, blood and urine culture were negative.

## Discussion

All five cases described above illustrate various forms of presentation of septic arthritis in patients with various medical backgrounds. History is clearly very important not only for diagnosis but to determine possible causative organism. For example, in Case 1, the history of recent sexual exposure gives rise to the suspicion of N.gonorrhea infection. The synovial and blood culture were negative, likely due to its nature as one of the fastidious organism [4]. Unfortunately, there was no Gram negative diplococcus seen during Gram stain examination. Urethral and rectal swab culture should have been sent to aid in making diagnosis [4]. The revision of diagnosis from reactive arthritis secondary to Chlamydia pneumoniae to septic arthritis was solely from clinical background as there is no laboratory test to distinguish between the two conditions.

In cases 2 and 3, the underlying organism was likely due to Staphylococcus aureus in view of history of skin infection and underlying rheumatoid arthritis respectively. An elderly and immunocompromised patient with septic arthritis is likely to grow Gram negative organism, as seen in Case 5. In the study by Dubost and colleagues, they found no changes in the distribution or organisms responsible for septic arthritis over a 20 year period [7].

History should also aim to look for the source of infection. In most cases, infection to the joint is through hematogenous spread; however history of skin break, penetrating trauma to the joint, prosthetic joint infection and history of intraarticular steroid injection need to be enquired [1, 8]. The affected joint is almost always warm, tender, erythematous and swollen with reduced range of movement.

The most frequently affected joint is the knee joint followed by hip, shoulder, ankle and wrist joint [1, 2, 7].

The diagnosis of septic arthritis can be difficult and up to today, there is still no single confirmatory test available. However, once suspected, antibiotic should be instituted. Gupta et al, in their study looking at morbidity and mortality between septic arthritis with a positive and negative synovial fluid culture, found similar picture in both groups of patient [9]. Thus, they conclude that it is justified to treat patients with high clinical suspicion of septic arthritis even in the absence of bacterial proof [9].

Once septic arthritis is suspected, synovial fluid should be sent for culture, Gram stain and white cell count. In the cases described, only one out of five cases yield positive culture. This could be due to earlier usage of antibiotic prior to synovial fluid aspiration. In another study by Dubost et al, they found that microorganisms were isolated from synovial fluid in 92% of patients with polyarticular septic arthritis and 95% of those with monoarticular septic arthritis [2]. Blood culture has lower positive rate with 76% in polyarticular involvement and 33% in monoarticular involvement [2].

Synovial fluid white cell count has also been used as a tool to diagnose septic arthritis, but it is still inconclusive. In a study by Coutlakis PJ in 2002 looking at 202 patients, he found those with synovial fluid WCC of more than 50 000/mm^3^ had a proven diagnosis of sepsis in 47% of cases and those with synovial fluid of more than 100,000/mm^3^ had a proven diagnosis in 77% of cases [10]. In another metanalysis by Margaretten et al, looking at 653 patients from 14 studies, they conclude that higher WCC count increase the likelihood of infection [11]. They concluded that counts of less than 25,000/mm^3^, more than 25,000/mm^3^, more than 50,000/mm^3^ and more than 100,000/mm^3^ gave a septic arthritis likehood ratio of 0.32, 2.9, 7.7 and 28.0 respectively [11]. However, they firmly suggest that this test should be use only in conjunction with clinical findings.

In our patients, unfortunately the reports from the lab mostly describe the number of white cells seen per high power field under the microscope. In Case 5, the only culture proven septic arthritis in this report had only 500/mm^3^ WCC seen, proving the point of the studies described above.

Peripheral white cell count, ESR and CRP has also been studied before in relation to diagnose septic arthritis, but none of these factors proved to be sensitive or specific [11-13]. A study by Soderquist et al showed that peripheral WBC, TNF alpha, IL-6, IL-8 and synovial WCC and glucoce had no predictive value in distinguishing reactive or infective arthritis [12]

Synovial fluid examination is also important to exclude crystal arthropathy, as illustrated in Case 4. Polyarticular gouthy arthritis may mimick polyarticular septic arthritis in clinical presentation associated with leucocytosis and raised inflammatory markers [12, 14]. In our patient, in view of chronic history to suggest rheumatoid arthritis, present of foci of infection and purulent synovial fluid aspirate, we were unable to exclude septic arthritis even after the diagnosis of gouthy arthritis. In fact, his infection was likely made worse by the underlying crystal arthropathy. However, we regret that the diagnosis was delayed and the patient should have been started with oral colchicine earlier.

Usage of PCR did not offer any advantage over bacterial culture in the diagnosis of staphylococcus and streptococcus infection [8, 13]. However, there was some success seen in the diagnosis of septic arthritis due to other organism such as Yersinia species, B. burgdoferi, Chlamydia species, N.gonorrhea and Ureaplasma species [8].

Imaging study is of limited use in joint infection. Radiograph is only useful to exclude osteomyelitis and ultrasound may be used to diagnose effusion in clinically distorted joints such as in rheumatoid arthritis [15].

As far as treatment is concerned, the appropriate antibiotic depends on suspected organism and local sensitivity and resistant pattern. So far, there is still no consensus or guideline on duration of antibiotic usage for treatment of septic arthritis. However, parenteral antibiotic for 2 to 4 weeks followed by 4 to 8 weeks of oral antibiotic has been recommended [16, 17].

Besides antibiotic, issue on medical versus surgical drainage is also controversial. There is no large randomized controlled trial to compare between these methods. However, a meta analysis by Broy and Schmid found that needle aspiration resulted in significantly greater percentage of good outcomes relative to surgery (73.7 versus 55.9%) as well as significantly higher mortality (5.6% versus 2.1%) [18].

Manadan and Block, in their review suggest daily arthrocentesis together with appropriate antibiotic in uncomplicated septic arthritis [19]. They suggest that surgical intervention to be reserved for complicated cases such as those with prosthetic joints or soft tissue extension, joints that are poorly accessible to needle aspiration such as hip joint and in cases with failed resolution of sepsis with daily arthrocentesis [19].

Despite antibiotic era and better access to medical facilities, the mortality and morbidity from septic arthritis is still high. Dubost found up to 30% mortality rate in patients with polyarticular septic arthritis either due to the infection or as a consequence of the disease [2]. In another study, Goldernberg and Cohen found almost 50% of patients with septic arthritis have significant sequelae of decreased range of movement or chronic pain [20] . Poor prognosis include infection of shoulder or hip, elderly more than 60 years old, underlying rheumatoid arthritis, delay in instituiting therapy and positive culture after seven days of appropriate therapy [20].

In summary, septic arthritis is a rheumatological emergency, diagnosed based on clinical ground supported by synovial fluid examination. Prompt treatment with appropriate antibiotic and synovial drainage are mandatory once septic arthritis is suspected to prevent long term complications.
